# Serum metabolic signatures of schizophrenia patients complicated with hepatitis B virus infection: A ^1^H NMR-based metabolomics study

**DOI:** 10.3389/fpsyt.2022.998709

**Published:** 2022-12-21

**Authors:** Caigui Lin, Qing Hu, Jiyang Dong, Zhiliang Wei, Jie Li, Zhong Chen

**Affiliations:** ^1^Fujian Provincial Key Laboratory for Plasma and Magnetic Resonance, Department of Electronic Science, Xiamen University, Xiamen, Fujian, China; ^2^National Institute for Data Science in Health and Medicine, Xiamen University, Xiamen, Fujian, China; ^3^Xiamen Xianyue Hospital, Xiamen, Fujian, China; ^4^Department of Radiology, Johns Hopkins University, Baltimore, MD, United States; ^5^Department of Hepatobiliary Surgery, Zhongshan Hospital of Xiamen University, Xiamen, Fujian, China

**Keywords:** NMR, metabolomics, schizophrenia, hepatitis B virus, synergistic action

## Abstract

**Introduction:**

Schizophrenia (SZ) is a severe chronic mental disorder with increased risk of hepatitis B virus (HBV) infection, which is incurable currently and induces various negative emotions and psychological pressures in patients to exacerbate mental disorders. To facilitate the therapeutic design for SZ patients complicated with HBV infection (SZ + HBV), it is helpful to first elucidate the metabolic perturbations in SZ + HBV patients.

**Methods:**

In this study, metabolic profiles of the serum samples from four groups of participants comprising healthy controls (HC, *n* = 72), HBV infection (*n* = 52), SZ patients (*n* = 37), and SZ + HBV (*n* = 41) patients were investigated using a high-resolution ^1^H NMR-based metabolomics approach.

**Results and discussion:**

Distinguishable metabolic profiles were found in the four groups. In comparison with HC, HBV infection induced increased levels of citrate and succinate to perturbate the tricarboxylic acid cycle and succinate-related pathways. Similar to SZ cases, SZ + HBV patients exhibited decreased glucose but increased citrate, pyruvate, and lactate, suggesting the occurrence of disturbance in glucose metabolism. Moreover, in comparison with HC, several serum amino acid levels in SZ + HBV patients were significantly altered. Our findings suggest that Warburg effect, energy metabolism disorders, neurotransmitter metabolism abnormalities, mitochondrial dysfunction and several disturbed pathways in relation to tyrosine and choline appear to play specific and central roles in the pathophysiology of SZ + HBV. Apart from replicating metabolic alterations induced by SZ and HBV separately (e.g., in energy metabolism and Warburg effect), the specific metabolic abnormalities in the SZ + HBV group (e.g., several tyrosine- and choline-related pathways) highlighted the existence of a synergistic action between SZ and HBV pathologies. Current study revealed the metabolic alterations specific to the interaction between SZ and HBV pathologies, and may open important perspectives for designing precise therapies for SZ + HBV patients beyond the simple combination of two individual treatments.

## Introduction

Schizophrenia (SZ) is a severe chronic mental disorder with symptomatic onset in early adulthood and persists throughout the whole lifespan (1). It affects approximately 0.5–1.0% of the world population with high complexity and heritability (2). Despite of the high prevalence, there is a lack of objective criteria to diagnose SZ, for which the current practice is interview with the suspected subjects or their custodians and is limited by the communication ability of subjects (e.g., in young children or neonates). In literature, genetic factors, environmental factors (e.g., living environment, drug abuse, and childhood trauma), and gene-environment interactions are reported to largely contribute to the liability of this disease (3).

Hepatitis B virus (HBV) infection is another worldwide health problem affecting 350 million people worldwide and causing 600,000 deaths annually by inducing hepatitis B-related liver diseases (4), which typically progress from acute infection to chronic hepatitis B, then to liver fibrosis and liver cirrhosis, and finally to hepatocellular carcinoma (HCC). HBV is responsible for more than half of the world’s HCC cases, which is amongst the three major causes of death in Asia and Africa. Up to now, the available treatments cannot eliminate the HBV completely from the body. The incurable nature of HBV often induces various negative emotions, e.g., depression and anxiety. Moreover, the misunderstanding of the infectivity of HBV further increase the psychological pressure on HBV-infected patients, thus promoting the development of mental disorders (5).

A series of previous studies have demonstrated that people with severe mental disorders are at increased risk for HBV infection (6–9). The increased prevalence is primarily attributed to the simple fact that those with severe mental disorders are more likely to engage themselves in risky sexual behavior, in abuses of psychoactive substances and injective drugs, which are the major means of HBV transmission (9). More recent report revealed that persons with mental disorders were at a higher risk than the general population for the development of comorbid conditions (10), including substance use disorder, metabolic disease and infectious disease. Therefore, the SZ cases complicated with HBV infection are not rare. However, the potential substance metabolism of synergistic action between SZ and HBV pathologies remains to be investigated, which is important for the targeted therapy in SZ + HBV patients.

Metabolomics, which focuses on small molecules in biological samples (e.g., cell, tissue, blood, urine, and cerebrospinal fluid), constitutes a fast technique to provide instantaneous snapshot of biochemical profiles, understand impairment of biochemical pathways, and delineate molecular-level mechanism of disease pathology (11). The high-resolution proton nuclear magnetic resonance (NMR) is one of the leading analytical approaches to metabolomics (12). NMR offers several unique advantages over other metabolomic platforms, such as non-destructive, unbiased, easily automatable, exceptionally reproducible, requires little to no sample preparation, has no need for chemical derivatization, and is the “gold standard” for the identification of novel compounds. NMR-based metabolomics has been applied to nearly every scientific field, including biomedicine, biomarker discovery and medical diagnosis, drug discovery, environmental science, agriculture, nutrition, food science, plant science, and renewable energy. NMR-based metabolomics has been widely used to reveal diagnostic biomarkers and biochemical pathways in SZ and HBV studies (13, 14). In this study, the ^1^H NMR-based metabolomics approach (15–17) will be utilized to profile the metabolome of HBV infection, SZ and SZ + HBV cases.

## Materials and methods

### Participants

The protocol of this study was reviewed and approved by the ethical committees of Xiamen University, Xiamen Zhongshan Hospital, and Xiamen Xianyue Hospital. All recruited participants were informed of the aims of the study, and informed consent was written and obtained from all participants or their authorized representatives. In this study, a total of 202 participants were recruited from October 2017 to November 2019 in Xiamen Xianyue Hospital and Xiamen Zhongshan Hospital.

The general inclusion criteria for all participants were as follows: (i) 18–65 years old; (ii) with read and write ability; (iii) no HBV vaccination. Participants with severe or unstable general medical conditions and participants in pregnancy or post-partum period will be excluded.

All SZ patients were diagnosed by trained psychiatrists referring to the detailed medical and psychiatric histories, including the Structured Clinical Interview for Diagnostic and Statistical Manual of Mental Disorders (DSM-IV). Specific inclusion criteria were as follows: (i) diagnosis of DSM-IV SZ or schizoaffective disorder; (ii) at least 1 year of follow-up. Patients with suicide risk, with diagnosis of a current substance use disorder (except nicotine dependence), or with mood-stabilizer treatment in the recent 1 month will be excluded.

Hepatitis B virus infection subjects were diagnosed by positive serum surface antigen of HBV (HBsAg). Subjects with HBV infection for more than 6 months will be included and those complicated with other liver diseases (e.g., hepatitis C virus, HCC, autoimmune hepatitis, and alcoholic liver disease) will be excluded.

SZ + HBV patients were subject to all the aforementioned inclusion and exclusion criterions. Healthy controls (HCs) met the following inclusion criteria: (i) no current or lifetime axis I psychiatric diagnosis; (ii) absence of known family member (checked up to second-degree relatives) with history of psychosis, mood disorders, or suicide. The HC group was age, gender, and risk factors matched in comparison with other groups.

After the screening, we have *n* = 72 for HC group (healthy controls), *n* = 52 for HBV group (HBV infection subjects), *n* = 37 for SZ group (SZ patients), and *n* = 41 for SZ + HBV group (SZ patients complicated with HBV infection).

### Sample collection

All blood samples were collected in the morning, 12 h after the last meal of the previous day (fasting conditions). Blood samples were collected into anticoagulant-free tubes and centrifuged at 1000 × *g* for 10 min to obtain blood serum and stored at –80°C. Blood sample of each participant was divided into two parts, one part for clinical biochemical analyses and the other for metabolomics analyses.

### Serum biochemical measurements

Clinical biochemical analyses of serum were performed based on photoelectron colorimetric detection principle using Beckman Coulter UniCel DxC 600 (Beckman Coulter, California, CA, USA). A total of 18 biochemical markers corresponding to glucose, lipid profile, liver function, and kidney function etc. were determined. All parameters are expressed as mean ± standard deviation.

### Sample preparation for NMR spectroscopy

After melting in ice, 400 μL serum sample was mixed with 200 μL deuterated phosphate-buffered solution (50 mM K_2_HPO_4_/NaH_2_PO_4_, pH 7.4, 0.9% NaCl, 99.9% D_2_O). After vortex and centrifugation (6700 × *g*, 4°C, 10 min), 500 μL supernatant was transferred into a 5 mm NMR tube and stored at 4°C before NMR analysis.

### NMR experiments

The ^1^H NMR experiments were performed using a Bruker NMR system (Bruker Biospin, Karlsruhe, Germany) operating at 850 MHz and 298 K temperature. The NMR spectra of serum samples were acquired by Carr-Purcell-Meiboom-Gill (CPMG) sequence (recycle delay∼π/2∼[τ∼π∼τ]_*n*_∼acquisition). The acquisition parameters were set as following: free relaxation delay of 4 s, acquisition time of 1.92 s, spectral width of 20 ppm, data point of 64 K, and 32 averages. This NMR sequence has been optimized and used in previous works (16).

### Preprocessing of NMR spectra

The acquired ^1^H NMR spectra were preprocessed using the MestReNova (v.8.1.2, Mestrelab Research S.L., La Coruña, Spain). All free induction decay signals were multiplied by an exponential function (line-broadening factor of 1.0 Hz) prior to Fourier transformation. Then, phase and baseline corrections were performed. For peak alignment, the left split of the doublet from lactate was set to 1.336 ppm as a chemical-shift reference. The spectra over the ranges of 0.5–9.0 ppm (excluding water resonance from 4.70 to 5.05 ppm and peak-free regions) were binned into bucket tables using dynamic adaptive binning method (18) and normalized using the probabilistic quotient normalization (PQN) method (19).

### Multivariate and univariate statistical data analyses

The preprocessed NMR data were imported into SIMCA software (version 14.1, Umetrics, Umeå, Sweden) for multivariate analysis, including unsupervised principal component analysis (PCA), supervised partial least-squares discriminant analysis (PLS-DA) and supervised orthogonal partial least-squares discriminant analysis (OPLS-DA). Unit-variance scaling was performed and then PCA analyses were applied to provide an overview of statistical trends and potential outliers. Then, PLS-DA and OPLS-DA were performed to reveal group-level metabolic differences. Comparisons between every two groups were carried out by using the OPLS-DA model. A 7-fold cross-validation and permutation test (200 permutations) followed by a cross-validation analysis of variance (CV-ANOVA) were performed for model validations.

The relative concentration of each metabolite was quantified by the integral over corresponding spectral range in reference to the internal standards. Peaks with least overlapping were picked out for quantifications of corresponding metabolites. For each metabolite, the fold change in concentration was calculated as the ratio of average concentrations between two different groups. A larger fold change indicates a severer metabolic perturbation. Transformed *P*-values from Student’s *t*-test were used to denote the significant level of metabolite differences. The fold change and *P*-values were used for univariate statistical analysis.

### Identification of characteristic metabolites

In this study, the results of multivariate and univariate statistical analyses were presented and visualized with four-dimensional enhanced volcano plots (16, 17). For the volcano plot, each circle represents a particular metabolite; the *x*-axis denotes log_2_(fold change); the *y*-axis denotes –log_10_(*P*-value); the values of variable importance projection (VIP) and absolute correlation coefficient (| r |) obtained from the OPLS-DA model were displayed with circles in different sizes and colors. In the plot, VIP values were categorized into three levels (VIP > 1.0, 1.0 ≥ VIP > 0.7 and VIP ≤ 0.7) and represented with three circle diameters (larger circle for higher VIP value). Circle colors varying from blue to red indicate the | r | values from 0 to 1 (a warmer circle color corresponds to a higher | r | value). In this study, characteristic metabolites were determined using the following criteria: *P*-value < 0.05, VIP > 1.0, and | r | > 0.4 (referred to the critical value table of correlation coefficient). Typically, characteristic metabolites appear as large circles in warm color on the upper left or upper right regions of the volcano plot.

### Metabolite set enrichment analysis

In order to investigate primary metabolic pathways perturbed in different groups, metabolite set enrichment analysis (MSEA) was performed using the MetaboAnalyst 5.0 software^1^ (20). Relevant analyses will follow the reported procedures (17). Briefly, the concentration table of identified characteristic metabolites from each group comparison (HBV vs. HC, SZ vs. HC, and SZ + HBV vs. HC) will be input for MSEA to visualize the altered metabolic pathways. Then, the top 20 perturbed pathways were sought out for each group comparison, the result highlighted a set of 37 different pathways collectively. Finally, the importance level of each pathway (smaller *P*-values correspond to higher importance levels) for each group comparison was summarized and exported to HemI (heatmap illustrator) for clustering analyses.

## Results

### Basic clinical characteristics

The metadata of the participants (including age, gender, blood lipid, blood glucose, liver function, and kidney function test results) are summarized in Table 1. In this study, no significant difference was found in age or gender between HBV, SZ, SZ + HBV groups, and HC group (all *P* > 0.05). However, we found the significantly different age between the SZ and SZ + HBV groups (*P* < 0.01). To determine if age confounds disease (SZ and HBV), a multivariate statistical model was built on the data, the analysis results (Supplementary Figure 1) indicated that there was no significant confounding effect between age and disease. Compared with the HC group, HBV group had significantly lower concentrations of γ-glutamyltranspeptidase (γ-GT) (*P* < 0.05), albumin (ALB) (*P* < 0.01), triglycerides (TG) (*P* < 0.05) and uric acid (UA) (*P* < 0.05); SZ group had significantly lower levels of total protein (TP), albumin (ALB), apolipoprotein (ApoA), glucose (Glu) and uera (all *P* < 0.01); SZ + HBV group had significantly lower levels of γ-glutamyltranspeptidase (γ-GT), total protein (TP), albumin (ALB), apolipoprotein (ApoA), glucose (Glu) and uera (all *P* < 0.01). These results implicate that liver dysfunction, kidney dysfunction, and metabolic disorder of blood lipid are common features for HBV infection subjects, SZ patients, and SZ + HBV patients. By contrast, blood glucose change is more specific to SZ, as observed in both SZ and SZ + HBV patients.

**TABLE 1:** Basic clinical and biochemical characteristics in different groups.

Parameters	HC (*n* = 72)	HBV (*n* = 52)	SZ (*n* = 37)	SZ + HBV (*n* = 41)
Gender (M/F)	38/34	26/26	19/18	24/17
Age (years)	36.74 ± 10.19	37.08 ± 10.47	34.95 ± 10.09	44.59 ± 10.21
ALT (U/L)	21.74 ± 13.59	24.45 ± 13.60	21.55 ± 15.62	23.23 ± 12.26
AST (U/L)	21.17 ± 6.19	22.67 ± 5.70	21.30 ± 7.80	23.54 ± 9.70
γ-GT (U/L)	29.60 ± 26.19	18.79 ± 9.67*	23.52 ± 17.89	19.92 ± 12.00**
ALP (U/L)	72.14 ± 20.15	69.84 ± 19.03	67.14 ± 18.30	74.59 ± 16.22
TBIL (μmol/L)	14.91 ± 4.99	14.55 ± 7.25	13.10 ± 5.73	12.79 ± 5.95
DBIL (μmol/L)	2.50 ± 0.80	2.56 ± 1.24	2.27 ± 0.97	2.26 ± 1.00
TP (g/L)	74.28 ± 3.86	73.04 ± 4.69	67.17 ± 5.32**	68.60 ± 3.87**
ALB (g/L)	47.65 ± 3.25	45.13 ± 3.44**	43.86 ± 3.64**	41.88 ± 3.57**
TG (mmol/L)	1.58 ± 0.77	1.01 ± 0.30*	1.60 ± 0.91	1.44 ± 0.62
TC (mmol/L)	4.99 ± 0.84	4.88 ± 0.75	4.85 ± 0.92	5.02 ± 1.04
HDL-C (mmol/L)	1.34 ± 0.29	1.40 ± 0.32	1.26 ± 0.34	1.23 ± 0.28
LDL-C (mmol/L)	2.83 ± 0.63	3.04 ± 0.78	2.78 ± 0.60	2.96 ± 0.70
ApoA (g/L)	1.20 ± 0.20	1.32 ± 0.23	1.05 ± 0.18**	1.03 ± 0.17**
ApoB (g/L)	0.83 ± 0.19	0.87 ± 0.18	0.84 ± 0.19	0.84 ± 0.19
Glu (mmol/L)	5.32 ± 0.71	5.16 ± 0.47	4.75 ± 0.59**	4.62 ± 0.57**
CREA (μmol/L)	65.43 ± 12.49	68.75 ± 18.56	66.35 ± 11.72	67.02 ± 12.24
Urea (mmol/L)	4.44 ± 0.99	4.38 ± 0.96	3.41 ± 0.93**	3.58 ± 0.95**
UA (μmol/L)	394.33 ± 103.45	342.45 ± 76.39*	407.86 ± 89.77	380.66 ± 90.13

Data are presented as mean ± standard deviation. HC, healthy controls; HBV, HBV infection patients; SZ, SZ patients; SZ + HBV, SZ complicated with HBV infection patients; M, male; F, female; ALT, alanine aminotransferase; AST, aspartate aminotransferase; γ-GT, γ-glutamyltranspeptidase; ALP, alkaline phosphatase; TBIL, total bilirubin; DBIL, direct bilirubin; TP, total protein; ALB, albumin; TG, triglycerides; TC, total cholesterol; HDL, high density lipoprotein cholesterol; LDL-C, low density lipoprotein cholesterol; ApoA, apolipoprotein A; ApoB, apolipoprotein B; Glu, glucose; CREA, creatinine; UA, uric acid. Student’s *t*-test was used for comparisons: *indicates *P* < 0.05 when compared with HC group; **indicates *P* < 0.01 when compared with HC group.

### ^1^H NMR spectral characteristics

**FIGURE 1:**
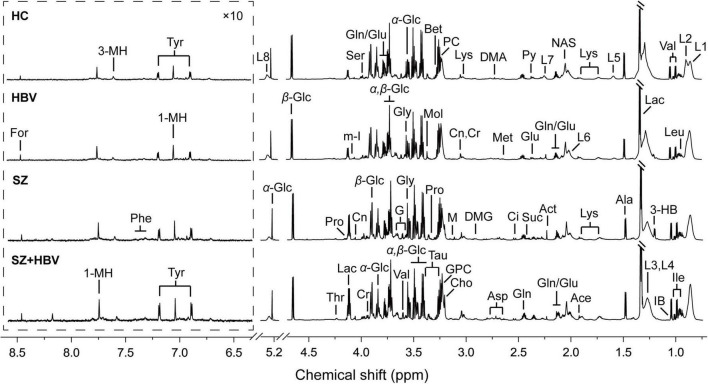
Typical metabolic profiles revealed by ^1^H NMR spectra at 850 MHz. Assignments were made as labeled for each group. Spectral regions from 6.3 to 8.7 ppm were displayed with a different scale for visual convenience. 1-MH, 1-methylhistidine; 3-HB, 3-hydroxybutyrate; 3-MH, 3-methylhistidine; Ace, acetate; Act, acetone; Ala, alanine; Asp, aspartate; Bet, betaine; Ch, choline; Ci, citrate; Cr, creatine; Cn, creatinine; DMA, dimethylamine; DMG, *N,N*-dimethylglycine; For, formate; G, glycerol; Glu, glutamate; Gln, glutamine; GPC, glycerophosphocholine; Gly, glycine; IB, isobutyrate; Ile, isoleucine; L1, LDL, CH_3_-(CH_2_)_*n*_-; L2, VLDL, CH_3_-(CH_2_)_*n*_-; L3, LDL, CH_3_-(CH_2_)_*n*_-; L4, VLDL, CH_3_-(CH_2_)_*n*_-; L5, VLDL, –CH_2_-CH_2_-C = O; L6, lipid, –CH_2_-CH = CH-; L7, lipid, –CH_2_-C = O; L8, lipid, –CH = CH-; Lac, lactate; Leu, leucine; Lys, lysine; M, malonate; Met, methionine; *m*-I, *myo*-Inositol; Mol, methanol; NAS, *N*-Actyl-glycoprotein signals; Phe, phenylalanine; PC, phosphocholine; Pro, proline; Py, pyruvate; Ser, serine; Suc, succinate; Tau, taurine; Thr, threonine; Tyr, tyrosine; Val, valine; α-Glc, α-glucose; β-Glc, β-glucose.

Typical ^1^H NMR spectra obtained from all four experimental groups (i.e., HC, HBV, SZ, and SZ + HBV groups) are shown in Figure 1. Referring to published literature (21, 22) and public metabolite databases (Human Metabolome Database, HMDB),^2^ the observed spectral peaks were assigned to 50 metabolites, including amino acids, organic acids, lipids and intermediates in energy metabolism. A complete list of metabolite assignments is summarized as Table 2. Inspection of ^1^H NMR spectra reveals a few metabolic differences, e.g., elevated concentrations of lactate and tyrosine in SZ + HBV groups when compared to HC group.

**TABLE 2:** Metabolite assignments of serum nuclear magnetic resonance (NMR) data.

Abbr.^a^	Metabolites	^1^H Shift (multiplicity^b^)	Moiety
1-MH	1-Methylhistidine	7.04(s); 7.74(s)	CH(2); CH(4)
3-HB	3-Hydroxybutyrate	1.20(d); 2.31(m); 2.41(m); 4.16(m)	CH_3_; α-CH; α-CH′; CH
3-MH	3-Methylhistidine	7.01(s); 7.61(s)	H_2_; H_4_
Ace	Acetate	1.92(s)	CH_3_
Act	Acetone	2.23(s)	CH_3_
Ala	Alanine	1.48(d)	CH_3_
Asp	Aspartate	2.67(dd); 2.82(dd); 3.90(dd)	β-CH; β-CH′; α-CH
Bet	Betaine	3.27(s); 3.89(s)	CH_3_; CH_2_
Ch	Choline	3.20(s)	CH_3_
Ci	Citrate	2.54(d), 2.69(d)	CH_2_; CH′_2_
Cr	Creatine	3.03(s); 3.93(s)	CH_3_; CH_2_
Cn	Creatinine	3.05(s); 4.06(s)	CH_3_; CH_2_
DMA	Dimethylamine	2.72(s)	CH_3_
DMG	*N,N*-Dimethylglycine	2.93(s); 3.73(s)	CH_3_; CH_2_
For	Formate	8.46(s)	CH
G	Glycerol	3.57(m); 3.65(m); 3.79(m)	CH_2_; CH′_2_; CH
Glu	Glutamate	2.05(m); 2.13(m); 2.35(m); 3.78(dd)	β-CH; β-CH′; γ-CH_2_; α-CH
Gln	Glutamine	2.14(m); 2.46(m); 3.78(t)	β-CH2; γ-CH_2_; α-CH
GPC	Glycerophosphocholine	3.23(s); 3.68(m); 3.96(m); 4.33(m)	CH_3_; N-CH_2_ and HO-CH_2_; CH and O-CH_2_; P-O-CH_2_
Gly	Glycine	3.56(s)	CH_2_
IB	Isobutyrate	1.07(d)	CH_3_
Ile	Isoleucine	0.94(t); 1.01(d)	δ-CH_3_; β-CH_3_
L1	LDL	0.86(br)	CH_3_-(CH_2_)_*n*_-
L2	VLDL	0.89(br)	CH_3_-(CH_2_)_*n*_-
L3	LDL	1.27(br)	CH_3_-(CH_2_)_*n*_-
L4	VLDL	1.30(br)	CH_3_-(CH_2_)_*n*_-
L5	VLDL	1.57(br)	–CH_2_-CH_2_-C = O
L6	Lipid	2.01(br)	–CH_2_-CH = CH-
L7	Lipid	2.23(br)	–CH_2_-C = O
L8	Lipid	5.31(br)	–CH = CH-
Lac	Lactate	1.33(d); 4.11(q)	CH_3_; CH
Leu	Leucine	0.96(t); 1.70(m)	CH_3_; CH_2_ and γ-CH
Lys	Lysine	1.73(m); 1.91(m); 3.02(t)	δ-CH_2_; β-CH_2_; ε-CH_2_
M	Malonate	3.15(s)	CH_2_
Met	Methionine	2.14(s); 2.16(m); 2.64(t); 3.86(t)	*S*-CH_3_; β-CH_2_; *S*-CH_2_; α-CH
*m*-I	*myo*-Inositol	3.28(t); 3.54(dd); 3.63(t); 4.07(t)	CH(2); CH(4, 6); CH(1, 3); CH(5)
Mol	Methanol	3.37(s)	CH_3_
NAS	*N*-Actyl-glycoprotein signals	2.05(s)	CH_3_
Phe	Phenylalanine	4.00(m); 7.33(d); 7.38(t); 7.43(m)	α-CH; *o*-CH; *p*-CH; *m*-CH
PC	Phosphocholine	3.22(s); 3.59(m); 4.17(m)	CH_3_; N-CH_2_; O-CH_2_
Pro	Proline	2.00(m); 2.08(m); 3.34(m); 3.43(m); 4.14(t)	γ-CH_2_; β-CH_2_; δ-CH_2_; δ-CH_2_; α-CH
Py	Pyruvate	2.37(s)	CH_3_
Ser	Serine	3.83(m); 3.96(m)	CH; CH_2_
Suc	Succinate	2.41(s)	CH
Tau	Taurine	3.27(t); 3.43(t)	CH_2_SO_3_; NCH_2_
Thr	Threonine	1.33(d); 3.59(d); 4.25(m)	CH_3_; α-CH; β-CH
Tyr	Tyrosine	6.89(d); 7.19(d)	*m*-CH; *o*-CH
Val	Valine	0.99(d); 1.04(d)	γ-CH_3_; γ-CH′_3_
α-Glc	α-Glucose	3.42(t); 3.54(dd); 3.71(t); 3.74(m); 3.84(m); 5.24(d)	CH(4); CH(2); CH(3); CH(6); CH(5 and 6′); CH(1)
β-Glc	β-Glucose	3.25(dd); 3.41(t); 3.46(m); 3.49(t); 3.72(dd); 3.90(dd); 4.65(d)	CH(2); CH(4); CH(5); CH(3); CH(6); CH(6′); CH(1)

^a^Abbr.: the abbreviation system is the same as used in Figure 1. ^b^Multiplicity: s, singlet; d, doublet; t, triplet; q, quartet; dd, doublet of doublets; m, multiplet; br, broad resonance.

### Multivariate statistical data analyses

**FIGURE 2:**
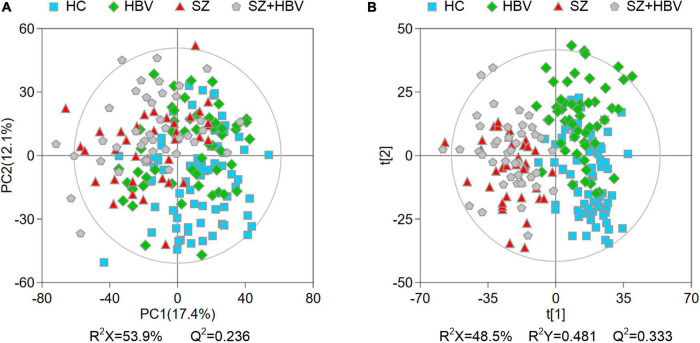
Principal component analysis (PCA) **(A)** and partial least-squares discriminant analysis (PLS-DA) **(B)** score plots of metabolic profiles in different groups. The outer ellipse represents the 95% confidence interval (Hotelling score).

**FIGURE 3:**
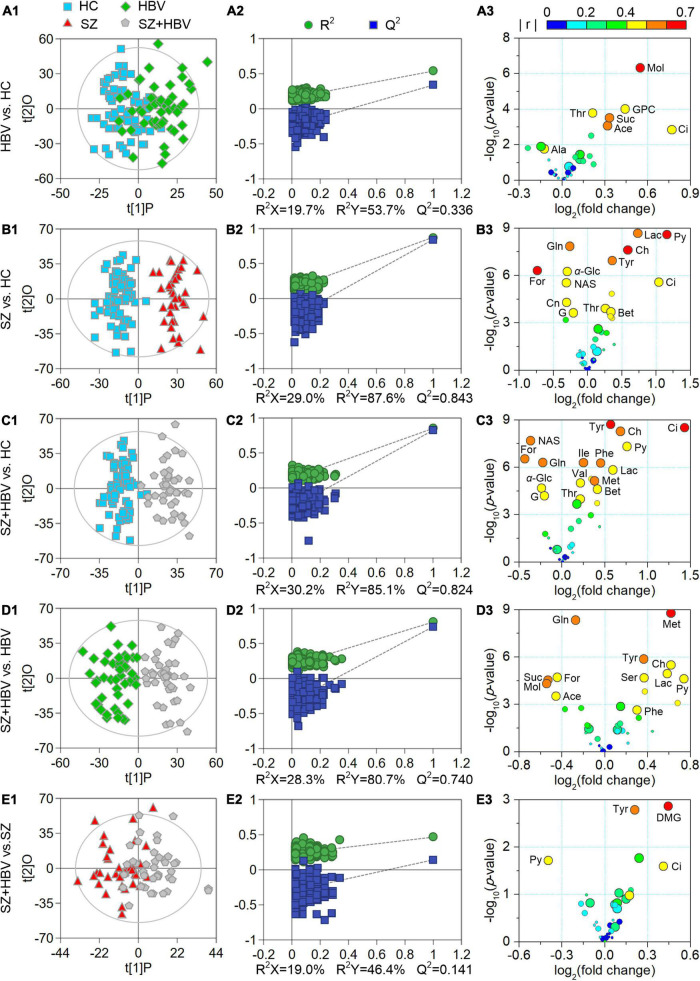
Score plots **(A1–E1)**, permutation test results **(A2–E2)**, and volcano plots **(A3–E3)** derived from different orthogonal partial least-squares discriminant analysis (OPLS-DA) models. In the volcano plots, circles in different sizes and colors denote variable importance projection (VIP) and | r | values, respectively. VIP values are categorized into three levels: VIP > 1.0, 1.0 ≥ VIP > 0.7, and VIP ≤ 0.7 represented by circles with decreasing diameters. Colors varying from blue to red indicate the | r | values from 0 to 1. Characteristic metabolites were identified by the following criteria: *P*-value < 0.05, VIP > 1.0, and | r | > 0.4.

Multivariate data analyses were used to explore the metabolic changes in HBV, SZ, SZ + HBV groups. The unsupervised PCA plot showed limited separation among different groups (Figure 2A). The supervised PLS-DA enhanced the overall separation in that there was clear separation between HC and any disease groups (HBV, SZ, or SZ + HBV) (Figure 2B). Note that SZ and SZ + HBV groups highly overlap on the PLS-DA score plot (Figure 2B), suggesting that metabolic disturbances induced by SZ plays a dominant role in the metabolic profiles of SZ + HBV patients. To evaluate the contribution of each metabolite in the separation of two groups, pair-wise comparisons (i.e., HBV vs. HC, SZ vs. HC, SZ + HBV vs. HC, SZ + HBV vs. HBV, and SZ + HBV vs. SZ) were carried out using the supervised OPLS-DA models (Figure 3). SZ + HBV and SZ groups are showed a slight overlap on the score plot of OPLS-DA model (Figure 3E1), the small R^2^X and Q^2^ values in the OPLS-DA comparing SZ + HBV and SZ groups further emphasized the similarity of metabolic profiles between these two groups and the dominant role of SZ pathology (Figure 3E2). However, distinctive separations were observed in the OPLS-DA models of HBV vs. HC, SZ vs. HC, SZ + HBV vs. HC, and SZ + HBV vs. HBV as well (Figures 3A1–D1). The permutation tests supported the validity of these OPLS-DA models, in which no over-fitting was observed (Figures 3A2–D2).

### Screening of characteristic metabolites and altered pathways

Enhanced volcano plots were used to screen characteristic metabolites that contributed to the group separation in pair-wise the OPLS-DA models (Figure 3). In comparison with HC, the HBV group showed increased levels of acetate, citrate, glycerophosphocholine, methanol, succinate, and threonine, together with decreased alanine. In comparison with HC, the SZ group exhibited increased levels of betaine, choline, citrate, lactate, pyruvate, threonine, and tyrosine along with decreased levels of creatinine, formate, glucose, glutamine, glycerol, and *N*-actyl-glycoprotein signals. By reference to HC, the SZ + HBV group replicated almost the metabolic alterations observed in SZ groups. In comparison with SZ, the SZ + HBV group was associated with higher citrate, tyrosine but lower pyruvate (three characteristic markers for SZ group referring to HC), suggesting a modulation by HBV on the activity of SZ-specific pathways. Moreover, the SZ + HBV group exhibited increased levels of isoleucine, methionine, phenylalanine, and valine, which have not been observed in SZ or HBV groups. It can therefore be inferred that the concurrent developments of SZ and HBV pathologies generates new alterations in the metabolic pathway(s), indicating a synergetic effect between SZ and HBV pathologies. Detailed fold change of the characteristic metabolites in different comparisons and the evaluating parameters of the pair-wise comparison models (including R^2^X, R^2^Y, Q^2^ and *P*-value) have been summarized in Table 3.

**TABLE 3:** Summary of the identified differential metabolites constructed from the comparison models.

Abbr.^a^		HBV vs. HC	SZ vs. HC	SZ + HBV vs. HC	SZ + HBV vs. HBV	SZ + HBV vs. SZ
	**R^2^X**	19.7%^b^	29.0%	30.2%	28.3%	19.0%
	**R^2^Y**	53.7%	87.6%	85.1%	80.7%	46.4%
	**Q^2^**	0.336	0.843	0.824	0.740	0.141
	***P***	<0.001	<0.001	<0.001	<0.001	0.024
Ace		1.25^c^	–	–	0.73	–
Ala		0.92	–	–	–	–
Bet		–	1.27	1.33	–	–
Ch		–	1.50	1.61	1.54	–
Ci		1.71	2.06	2.74	–	1.33
Cn		–	0.81	–	–	–
DMG		–	–	–	–	1.36
For		–	0.60	0.71	0.74	–
G		–	0.86	0.88	–	–
Gln		–	0.83	0.86	0.83	–
GPC		1.36	–	–	–	–
Ile		–	–	1.19	–	–
Lac		–	1.66	1.51	1.50	–
Met		–	–	1.30	1.54	–
Mol		1.46	–	–	0.69	–
NAS		–	0.81	0.78	–	–
Phe		–	–	1.37	1.23	–
Py		–	2.23	1.70	1.67	0.76
Ser		–	–	–	1.29	–
Suc		1.26	–	–	0.69	–
Thr		1.16	1.20	1.16	–	–
Tyr		–	1.28	1.48	1.29	1.16
Val		–	–	1.16	–	–
α-Glc		–	0.81	0.85	–	–

^a^The same abbreviation system as used in Figure 1 and Table 1. ^b^The evaluating parameters of the OPLS-DA models include R^2^X, R^2^Y, Q^2^, and P-value. ^c^The fold change values were calculated as the ratio of concentrations in two groups (HBV vs. HC, SZ vs. HC, SZ + HBV vs. HC, SZ + HBV vs. HBV, and SZ + HBV vs. SZ). Therefore, numbers greater and less than 1 represent increased and decreased concentrations in the former group as compared to the latter group. “-” Means non-significant difference between two groups.

A total number of 56 pathways were involved in the comparisons between HBV and HC, between SZ and HC, and between SZ + HBV and HC (i.e., HBV vs. HC, SZ vs. HC, and SZ + HBV vs. HC). Referencing to HC group, characteristic metabolites of HBV group are enriched in pathways including *tricarboxylic acid (TCA) cycle*, *retinol metabolism*, *threonine and 2-oxobutanoate degradation*, *glycine and serine metabolism*, and as well as *valine, leucine and isoleucine degradation*, while characteristic metabolites of SZ group are enriched in *transfer of acetyl groups into mitochondria*, *amino sugar metabolism*, *urea cycle*, *methionine metabolism and betaine metabolism*. The enriched metabolic pathways for SZ + HBV patients included *Warburg effect*, *TCA cycle*, *methionine metabolism*, *betaine metabolism*, and *ammonia recycling*.

### Synergistic action of SZ and HBV infection

**FIGURE 4:**
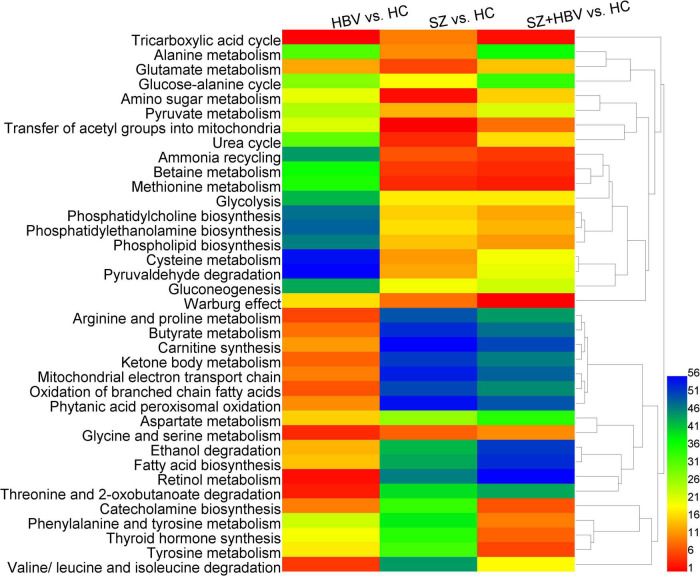
Heat-map of perturbed metabolic pathways. Columns represent different comparison models: HBV vs. HC, SZ vs. HC, and SZ + HBV vs. HC; and rows represent metabolic pathways, respectively. The significance order of metabolic pathways was color encoded (red representing the most important and blue the least important). The unsupervised hierarchical clustering of metabolic pathways reveals the relationship between different pathways (shorter branch corresponds to stronger correlation).

Figure 4 shows the heat-map and clustering result of ranks of the dysfunctional pathways.

Comparisons between HBV and HC suggested lower level of succinate in the HBV group, which was involved in *TCA cycle*, *arginine*, and *proline metabolism*, *butyrate metabolism*, *carnitine synthesis*, *ketone body metabolism*, *mitochondrial electron transport chain*, *oxidation of branched chain fatty acids*, and *phytanic acid peroxisomal oxidation* with very high importance and strong correlation. In the comparison between SZ and HC groups, there was not a significant change in succinate level. Accordingly, the corresponding pathways were associated with less importance in SZ and SZ + HBV patients.

Comparisons between SZ and HC suggested higher levels of betaine and choline in the SZ group, which were involved in *betaine metabolism*, and *methionine metabolism* with very high importance and strong correlation, and also alterations in glutamine and pyruvate, which were involved in *ammonia recycling*, *urea cycle*, *amino sugar metabolism*, and *glutamate metabolism* with very high importance.

Comparison between SZ + HBV and HC revealed increased betaine, choline and methionine in the SZ + HBV group, which were involved in *betaine metabolism* and *methionine metabolism* with high importance and strong correlation, and increased tyrosine, which was involved in *tyrosine metabolism*, *phenylalanine* and *tyrosine metabolism*, *thyroid hormone synthesis* and *catecholamine biosynthesis* with very high importance and strong correlation. By contrast, the tyrosine-related pathways were characterized by less importance in HBV infection or SZ patients. The SZ + HBV group also exhibited higher choline, which was involved in *phosphatidylcholine biosynthesis*, *phosphatidylethanolamine biosynthesis*, and *phospholipid biosynthesis* with high importance and strong correlation. These choline-related pathways were characterized by less importance in HBV infection or SZ patients. It is worthy of noting that the *TCA cycle* and *Warburg effect* related to citrate and pyruvate were associated with high importance in both SZ and HBV infection patients, and this importance became even higher in SZ + HBV patients.

Based on these results, metabolic alterations in SZ + HBV show more similarity to SZ than HBV. The succinate-related pathways are specific pathways for single HBV pathology; betaine- and choline-related pathways plays important roles in both SZ and SZ + HBV patients; citrate- and pyruvate-related pathways (i.e., *TCA cycle* and *Warburg effect*) are common pathways with perturbations in SZ, HBV infection, and SZ + HBV patients, in addition, importance became even higher in SZ + HBV patients (synergistic action). In particular, tyrosine- and choline-related pathways are specific to the mixed pathologies of SZ and HBV (synergistic action).

## Discussion

**FIGURE 5:**
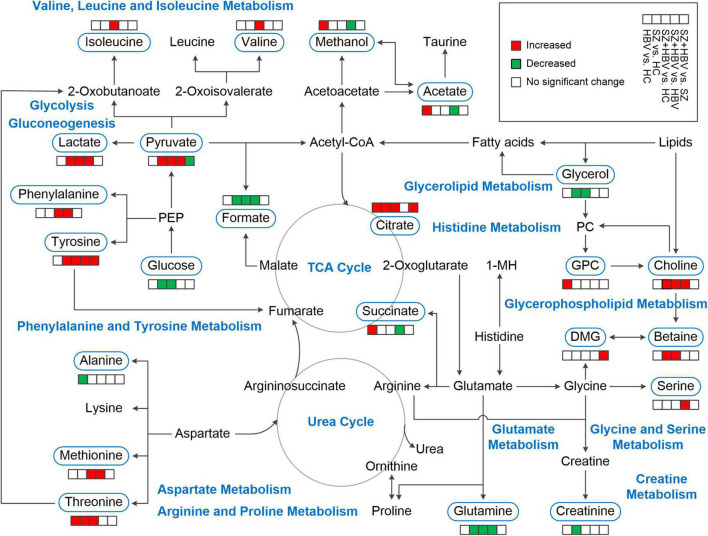
Schematic overview of metabolites and major metabolic pathways. 1-MH, 1-methylhistidine; GPC, glycerophosphocholine; PC, phosphocholine; PEP, phosphoenolpyruvate; TCA, tricarboxylic acid.

Our NMR-based metabolomics study revealed multifaceted metabolic alterations in SZ, HBV infection, and SZ + HBV patients (Figure 5). Apart from the characteristic metabolic markers for single pathology, we have identified the metabolic pathways specific to the mixed pathologies of SZ and HBV. For example, a single combination of individual SZ and HBV treatments may be insufficient for SZ + HBV patients, and a more comprehensive treatment taking the synergetic action into consideration will prove helpful in improving the final neurological outcome.

Acetate is the end product of lipid metabolism, the increased acetate in HBV infection subjects suggested an increase of lipid metabolism in response to liver injury. This finding agreements with previous investigations reporting increases of serum acetate levels in other liver diseases such as cirrhosis and HCC (23, 24). In HepG2.2.15 cells, which are popularly used in HBV studies, some enzymes regulating tricarboxylic acid (TCA) cycle were upregulated and intermediates in the TCA cycle, e.g., succinate, were increased to show increased activity of TCA cycle (25). The observed succinate and citrate in HBV infection subjects indicated a perturbation in TCA cycle, which was further supported by the pathway enrichment analyses that TCA cycle and mitochondrial electron transport chain were disturbed with high importance in HBV infection subjects. TCA hyperactivity along with mitochondrial dysfunction can result in the production of oxidative stress in hepatitis patients (26, 27).

Several hypothetical mechanistic model for SZ have been proposed in literature, including oxidative stress and inflammation hypothesis, polyunsaturated fatty acid metabolism hypothesis, membrane phospholipid hypothesis, energy metabolism disorder, glutamatergic hypothesis, and dopamine hypothesis (13). Current study shows consistency with the major conclusions in these studies, and therefore serves as an additional proof.

The membrane phospholipid hypothesis in relation to SZ was postulated by Horrobin (28). Normal neuronal phospholipid metabolism is required for the normal development of brain architecture and for neuronal functioning. Because of the central role of phospholipids, particularly in neurons, a phospholipid abnormality will inevitably lead to secondary abnormalities in neurotransmitters, ion channel, and cell signaling systems. Schwarz et al. (29) reported significant alternations in free fatty acids and phosphatidylcholine in gray and white matter of SZ patients. Fenton et al. (30) demonstrated that cell membrane abnormalities were associated with disordered phospholipids composition and metabolism in SZ pathogenesis. Choline is often considered as a marker for membrane phospholipid turnover. The higher levels of choline relevant to the phospholipid biosynthesis and phosphatidylcholine biosynthesis perturbation in SZ and SZ + HBV patients of our study is thought to reflect active membrane phospholipid breakdown, and may induce secondary abnormalities in neurotransmitters. Moreover, Li et al. (25) investigated the metabolic features of host cells infected with HBV and results showed that HBV infection up-regulated the phosphatidylcholine biosynthesis by activating choline kinase alpha (CHKA) which will lead to HBV-associated HCC. Note that suppressing phosphatidylcholine biosynthesis can inhibit HBV replication and expression. Choline-related pathways were not significantly perturbed in HBV infection patient when compared with HCs. However, alterations of choline-related pathways were prominent in the SZ + HBV patients, which may be attributed to the interplay between SZ and HBV pathology. Such an interplay tended to exacerbate the HBV replication and promote the conversion from HBV to HCC, requiring special attentions in the daily care and treatment of SZ + HBV patients.

Various metabolomics studies have highlighted that malfunction of glucose metabolism may be a causative factor for SZ (31–35). In adults, the brain accounts for 20% of the total body basal oxygen consumption and 25% of the total body glucose utilization. Glucose is the major substrate for aerobic metabolism, glycolysis coupled with the TCA cycle fully oxidizes glucose and supplies energy for the brain (36). Glucose metabolism homeostasis is fundamental to maintain normal brain functions. Our finding of lower glucose content in serum (with clinical biochemical analyses or NMR-based metabolomics analyses) is consistent with the previous report of downregulation in serum glucose (37), which may be a contributing factor to the chronic glucose deficiencies in the brain of SZ and SZ + HBV patients. As an important intermediate in glucose metabolism, pyruvate connects glycolysis and TCA. In our study, decreased glucose together with increased pyruvate, lactate, and citrate in SZ and SZ + HBV patients were attributed to increased glycolysis. Another possible cause for glucose reduction is elevated serum insulin, which promotes glucose uptake, as repeatedly reported in first-episode neuroleptic-naïve SZ patients (38, 39). A higher prevalence of glucose tolerance and insulin resistance in patients with first-episode SZ has been attributed to the abnormal glucose metabolism (40–42). It is presumed that elevated insulin may be responsible for normoglycemia or even relative hypoglycemia in SZ patients at an early stage of their first-episode (37). Moreover, additional glucose supply has been found to improve memory performance in SZ patients (43). Therefore, glucose supplement may serve as a promising adjuvant therapy for SZ or SZ + HBV patients.

Lactate is the end-product of glucose metabolism under anaerobic conditions, which may happen in brain due to insufficient nutrient and oxygen supply, e.g., in ischemic stroke or late stages of neurodegenerative diseases (44). Lactate shows potential to be a biomarker for SZ due to its involvement in bioenergetic pathways, which are known to be altered in SZ (45). Our finding of elevated lactate shows consistency with previous reports (45, 46). Elevation of serum lactate may suggest increased glycolysis for energy production in SZ and SZ + HBV patients. Another possible reason for increased lactate is oxidative stress-related mitochondrial dysfunction (47), the increase of oxidative stress (reactive oxygen species production) leads the cells toward the glycolytic way with lactate production (48). As one intermediate of the TCA cycle, increased level of citrate was also observed in SZ and SZ + HBV patients, indicating abnormalities in TCA cycle activity and mitochondrial dysfunction. Converging evidence indicates that mitochondrial dysfunction is linked with SZ (33, 49–51). Based on the metabolic pathway enrichment analyses, transfer of acetyl groups into mitochondria pathways were dramatically altered in SZ and SZ + HBV groups, which partly supported the notion of mitochondrial dysfunctions in SZ and SZ + HBV patients.

In our study, metabolites directly associated with energy metabolism contributed to the separation between SZ and HC, and between SZ + HBV and HC groups. The hyperactivity of energy metabolism may indicate an complementary process for inefficiency of brain circuitry in SZ and SZ + HBV patients (52). There was a hypothesis that the virus induced glycolysis over oxidative phosphorylation in a similar manner to the Warburg effect in cancer (53), and previous study reported that an increase in energy metabolism could be considered as an important characteristic during the progression of HBV-related liver cirrhosis (54). Taking all these results into consideration, we speculate that the mixed pathologies of SZ and HBV enhance the energetic metabolism and Warburg effect, accelerate the process from chronic HBV to liver cirrhosis. Therefore, it will be beneficial to monitor the liver condition of SZ + HBV patients to determine early intervention.

Disturbance of amino acid metabolism is a common metabolic feature in HBV or SZ patients. Since liver is a major organ for amino acid conversion, abnormality in protein metabolism occurs commonly in liver disease. Meng et al. (55) have reported that patients with chronic HBV and HBV-related liver cirrhosis had a higher protein oxidation rate. Therefore, we speculate that proteolysis is activated in SZ + HBV patients, leading to increased levels of amino acid in blood and protein deficiency. In comparison with all the other groups, the SZ + HBV group exhibited severer perturbations in various amino acids due to the co-development of two pathologies contributing to a change toward the same direction.

Tyrosine is the first product in phenylalanine catabolism, phenylalanine is hydroxylated to tyrosine by phenylalanine hydroxylase in liver and kidney. Dejong et al. (56) have reported that the conversion of phenylalanine to tyrosine is an exclusive function of the liver. HBV patients with liver dysfunction frequently present with increased blood phenylalanine and tyrosine levels (57). Thus, parallel accumulations of phenylalanine and tyrosine in serum suggested the possible loss of liver function in SZ + HBV patient. Moreover, Yang et al. (57) reported that the ratio of branched chain amino acids (BCAAs, i.e., valine, leucine, and isoleucine) to tyrosine could be used for the diagnosis, treatment selection, and prognosis of patients at different stages of HBV infection. Furthermore, the tyrosine concentrations in blood from patients with chronic severe HBV significantly increased (57). Numerous studies reported that tyrosine increased from no/mild fibrosis to severe fibrosis (58, 59). Specifically, in comparison with HC, HBV, and SZ patients, level of tyrosine in SZ + HBV patients was significantly higher. Our metabolic pathway enrichment analyses further indicated that tyrosine-related pathways, including tyrosine metabolism, phenylalanine and tyrosine metabolism, thyroid hormone synthesis, and catecholamine biosynthesis, were significantly disturbed in SZ + HBV patient with very high importance. Based on these consistent findings, alterations of tyrosine-related pathways are therefore considered as the most important feature for SZ + HBV patients. The interplay between SZ and HBV pathologies induces broader metabolic perturbations to potentially promote the associated major pathological processes.

Chen et al. (60) found that phenylalanine, tyrosine, and tryptophan biosynthesis was a differential metabolic pathway between the SZ patients with and without violence tendency. Moreover, an earlier study reported that tyrosine supplements increased aggressive behaviors in mice (61). Current finding of elevated tyrosine level in the SZ + HBV group (in comparison with HC or SZ) may highlight the extra importance of caring the violence risk in SZ + HBV patients.

Orešič et al. (62) found higher levels of phenylalanine, tyrosine, isoleucine, threonine, and methionine in serum of SZ patient, our observations align well with those finding. Being large neutral amino acids, phenylalanine, tyrosine, valine, leucine, and isoleucine travel across the blood-brain barrier into the brain through the same type of neutral amino acid transporter LAT-1 (63). Hence, for SZ + HBV patient, influx of phenylalanine and tyrosine in the brain could be reduced due to the competition with increased concentrations of valine and isoleucine. Phenylalanine and tyrosine are precursor of catecholamine (i.e., dopamine, epinephrine, and norepinephrine). The abnormal presence of phenylalanine and tyrosine in brain may subsequently result in imbalances of neurotransmitter biosynthesis in SZ + HBV patient. Consistently, our metabolic pathway enrichment analyses revealed that tyrosine-related pathways, including tyrosine metabolism, phenylalanine and tyrosine metabolism, thyroid hormone synthesis and catecholamine biosynthesis, were significantly disturbed in SZ + HBV patient with very high importance. Valine and isoleucine play important roles in stimulating insulin secretion, elevated levels of valine and isoleucine in SZ + HBV patient may result in elevated insulin, current finding aligns well with earlier reports of elevated insulin in first-episode neuroleptic-naïve SZ patients (38, 39).

Considerable evidences indicated that dysfunction of glutamine-glutamate cycle was involved in the pathophysiology of SZ, which contributed to the abnormalities in glutamatergic neurotransmission in SZ (64, 65). Altered glutamate and glutamine levels in the blood have been reported in SZ, but results were not consistent (66). The finding indicated that changes in peripheral glutamate and glutamine levels may occur in SZ, but the direction of change appears to be dependent on the duration of this disorder. More recently, a study found increased glutamine/glutamate ratio in patients with first-episode SZ and decreased glutamine/glutamate ratio in chronic SZ cases as compared to healthy controls (66). In our study, we found decreased level of glutamine in SZ and SZ + HBV patients, showing consistency with the previous studies reporting lower concentration of glutamine in SZ blood samples (67, 68) and decreased levels of the glutamine synthetase protein in post-mortem brain tissue from patients with chronic SZ (65, 69). The decreased glutamate will indicate the glutamine-glutamate cycle dysfunction in SZ and SZ + HBV patients. In current study, we did not observe a significant change in glutamate. And first-episode and chronic SZ patients were both recruited in our study, future study focusing on the glutamate and glutamine in staged SZ cohorts will further delineate the outcome of perturbations in glutamine-glutamate cycle.

In summary, current findings are overall consistent with previous HBV and SZ metabolomics studies. Results and conclusions in current study should be cautiously interpreted due to the following limitations. First, the pathological stages of SZ patients were heterogenous, including first-episode drug-naïve SZ patients, chronic SZ patients, and SZ patients after drug discontinuance. Note that this is partly due to our strict enrollment criterion as detailed in method section to control the other variabilities. Note that the coverage of heterogenous stages helps to avoid over interpretation of a specific SZ stage and to focus on the common features throughout the whole process of pathological developments. Second, primarily limited by the sample size, our current sample size will not support categorized analyses in subgroups, e.g., correlating the severity of SZ with metabolic alterations to gain insights into the specificity and sensitivity of biomarker metabolites. Further studies toward this direction will facilitate the understanding of association between pathogenic progression and metabolic pathway perturbations. Third, the sex difference on the metabolic alterations has not been systematically investigated. Both male and female participants were recruited in current study. But a much larger cohort will be required to ensure statistical power if the sex effect is focused. Thirdly, although we have controlled for the effects of current lifestyle, long-term effects of lifestyle-related factors (e.g., smoking, diet, and exercise) may not have been captured. Fourth, a limitation of our study is the lack of systematic education and socio-economic information. Our subjects in each group involved participants with variable education and socio-economic status. A dedicated epidemiological study covering a larger population will prove helpful in delineating the effects of education or socio-economic status on the pathogenic metabolism alterations. Finally, the current study focused on the NMR-based metabolomics analyses on metabolites with relatively small molecular weights. Multi-modality study together with proteomics or transcriptomics will prove helpful in validating the results and revealing underlying pathological mechanism with multiscale proofs.

## Conclusion

In this study, a ^1^H NMR-based metabolomics approach was used to investigate the metabolic perturbations of schizophrenia complicated by hepatitis B virus infection. To the best of our knowledge, current study is the first report to investigate the metabolic perturbations in SZ patients complicated with HBV infection. Our findings suggest that SZ + HBV patients share several common important metabolites with SZ and HBV infection patients. Moreover, several characteristic metabolites specific to SZ + HBV have been highlighted, revealing that Warburg effect, energy metabolism disorders, neurotransmitter metabolism abnormalities, mitochondrial dysfunction, and several disturbed pathways related to tyrosine and choline appear to play central roles in the pathophysiology of SZ + HBV. Moreover, energy metabolism together with tyrosine- and choline-related pathways play central roles in the synergistic action between SZ and HBV infection. These metabolic findings will promote the understanding of biological mechanisms underlying co-development of SZ and HBV pathologies, and facilitate future therapeutic designs. The metabolic basis associated with SZ + HBV suggests that metabolomics can be a powerful tool to provide clues for diagnosis, treatment, and follow-up of SZ + HBV, particularly considering the convenience, low cost, and patient comfort with collecting blood samples only.

## Data availability statement

The raw data supporting the conclusions of this article will be made available by the authors, without undue reservation.

## Ethics statement

Our work was carried out according to the Declaration of Helsinki. All participants provided written informed consent to allow use of their samples in these analyses. The protocol of this study was reviewed and approved by the Ethical Committees of Xiamen University, Xiamen Zhongshan Hospital, and Xiamen Xianyue Hospital (MEC-XY-2016-0028).

## Author contributions

CL, ZC, and JL conceived and designed the research. CL and QH performed the data collection and wrote the manuscript. CL and JD analyzed the data. JD, ZW, JL, and ZC critically revised the manuscript. All authors have read and approved the manuscript.

## Footnotes

1
http://www.metaboanalyst.ca/

2
http://www.hmdb.ca
