# Supplementary material for: Serum metabolic signatures of schizophrenia patients complicated with hepatitis B virus infection: A 1H NMR-based metabolomics study
Source: Front Psychiatry. 2022 Dec 21;13:998709. doi: 10.3389/fpsyt.2022.998709 (PMC9810819; doi:10.3389/fpsyt.2022.998709)
Supplement: Supplementary file 1 [file Data_Sheet_1.PDF]

Supplementary Files

In this study, there is no significant difference between HBV, SZ, SZ+HBV groups and healthy control group (all $P > 0.05$) in age. Interestingly, the metabolic profiles are similar between the SZ and SZ+HBV groups despite of the significantly different age.

To investigate the potential confounding effect of age on metabolism, we divided all samples into four groups according to age, and carried out the partial least-squares discriminant analysis (PLS-DA) with age as the response variable Y , the results are shown in **Supplementary Figure 1**. In the score plot (**Supplementary Figure 1A**), there was no obvious separation among the four groups. From the permutation test result (**Supplementary Figure 1B**), the small R^2X and Q^2 values further indicated the unavailability of this PLS-DA model and the effect of age on metabolic profile was not significant.

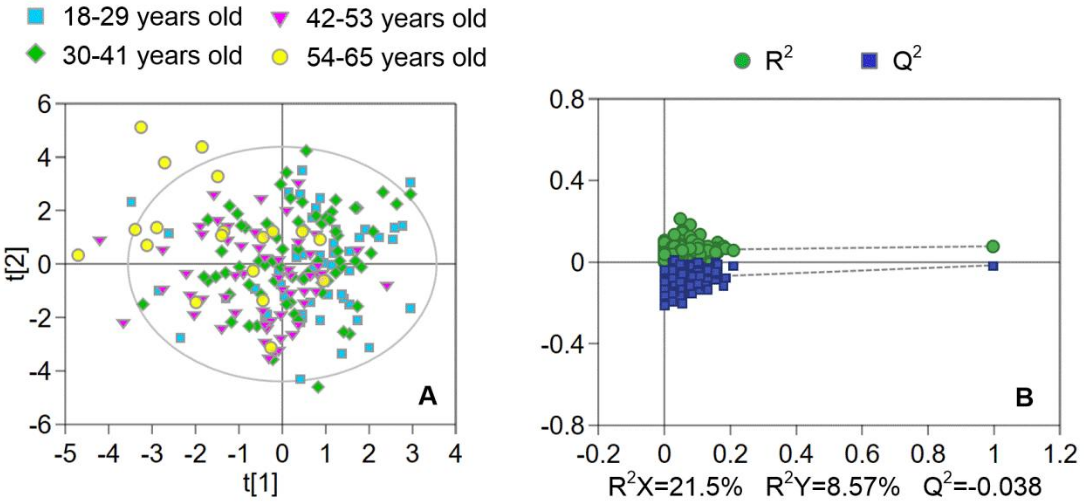

Supplementary Figure 1. The score plot (A) and permutation test result (B) derived from PLS-DA model.
